# Factors Influencing Depression Among Female Professional Caregivers as per Employment Type (Full-Time vs. Part-Time)

**DOI:** 10.3390/healthcare13172242

**Published:** 2025-09-08

**Authors:** Ji-Hyun Moon, Hye-Sun Jung

**Affiliations:** Department of Preventive Medicine, College of Medicine, The Catholic University of Korea, Seoul 06591, Republic of Korea; moonji@catholic.ac.kr

**Keywords:** professional caregivers, depression, employment type

## Abstract

**Background/Objectives:** South Korea is rapidly transitioning into a super-aged society, increasing the importance of care services to ensure the health and quality of life of older adults. Although the number of professional caregivers has steadily grown, these workers face a high risk of depression due to the emotional labor inherent to their roles. This study aimed to analyze factors influencing depression among female professional caregivers by employment type (full-time and part-time) and to explore policy and practical intervention strategies to promote their mental health. **Methods:** Using data from the Korea Occupational Safety and Health Agency, we selected 223 professional caregivers with at least 1 year of work experience. After excluding insincere responses and male participants, 217 participants were included in the final analysis: 121 full-time and 96 part-time professional caregivers. **Results**: We found that full-time workers experienced higher levels of depression when they lacked access to health and safety education and could not use paid leave. Part-time workers experienced high levels of depression when engaging in physical activity <3 days per week and when exposed to violence. **Conclusions**: Based on these findings, the study recommends regular and systematic health and safety education, the establishment of substitute worker support to facilitate paid leave use, promotion of physical activity through education and community programs, regular violence prevention education, and comprehensive support systems for victims. This study is significant in empirically identifying depression risk factors by employment type among female professional caregivers. Future research should include male professional caregivers and employ more advanced measurement tools and longitudinal designs.

## 1. Introduction

In 2023, the elderly population (≥65 years old) in South Korea was 9,500,000, constituting 18.4% of the total population of 51,558,800. The percentage of elderly individuals is predicted to continue increasing to 20.6% in 2025, 30.1% in 2035, and 40.1% in 2050 [1]. Consequently, South Korea is rapidly progressing toward a super-aged society, underscoring the importance of care services in supporting the physical and mental health of the elderly and ensuring their quality of life.

As of 2023, the cumulative number of individuals with a recognized long-term care grade was 1,097,913, accounting for 11.1% of the elderly population aged 65 years and older (9,858,810 individuals), which represents a 7.7% increase compared to the previous year [2]. The number of professional caregivers providing elderly care services is also increasing annually. In 2023, 482,229 professional caregivers were employed in South Korea, of which 93.6% (451,486 individuals) were female [3].

As professional caregivers provide care in the client’s living space, the job involves high levels of emotional labor and psychological tension, which may cause severe mental health problems in professional caregivers [4,5]. Work-related emotional burnout can increase mental health risk, including anxiety, depression, and suicidal ideation [6,7]. Among these issues, depression affects 25% of all workers and causes work loss, increasing economic costs [8]. According to previous studies, 15.5% of care workers who provide services to older adults and individuals with disabilities exhibited moderate to severe levels of depression, as indicated by a PHQ-9 score of 10 or higher [9]. In addition, among home care workers, 32.3% reported experiencing depressive symptoms, and 32.0% reported high levels of stress [10]. Furthermore, 26.2% of professional caregivers were found to be experiencing clinically significant depression [11]. These findings highlight the elevated risk of mental health problems among professional caregivers and underscore the necessity of targeted interventions to address their psychological well-being. Therefore, there is a need to identify the factors affecting depression in professional caregivers and prepare interventions for these factors.

One factor affecting depression is employment type, which can be categorized as full-time or part-time based on work hours. In 2023, 59.7% of professional caregivers in South Korea (287,791 individuals) were full-time [3]. Full-time labor provides economic stability owing to the fixed work time and income but also limits lifestyle flexibility and can lead to long work hours or overwork [4]. Part-time employment offers flexible working hours, which can facilitate a better work–life balance for individuals [12]. However, it may also contribute to increased stress levels due to economic disparities arising from lower wages, greater work intensity, and employment instability [13]. Such employment-related stress is closely linked to workers’ mental health. In full-time positions, stress is primarily associated with excessive workloads, whereas part-time workers are more likely to experience stress stemming from both mental and physical demands, as well as anxiety regarding their future [14]. All of these factors have been found to be associated with depression [14]. Notably, female part-time workers are reported to be 1.8 times more likely to experience depression compared to their full-time counterparts [15].

This study aims to identify the factors influencing depression among professional caregivers according to employment type and to provide evidence to support the development of mental health promotion interventions tailored to each group.

## 2. Materials and Methods

### 2.1. Data Collection

In this study, we used data collected from the ‘Survey on the State of Health and Safety for Caregivers and Health Survey to Prepare Health Protection Measures’ conducted by the Korea Occupational Safety and Health Agency [16].

A convenience sampling method was employed to recruit professional caregivers working at care institutions nationwide. Data were obtained between July and October 2022. The survey was conducted through the Korea Association of Caregivers and related organizations, and participation was limited to individuals who voluntarily consented after receiving an explanation of the study’s purpose and content. The URL for the online questionnaire was distributed, and the survey was conducted anonymously. This study was approved by the Institutional Review Board of the Catholic University of Korea (IRB approval number: MC23EASI0074), and strict confidentiality and anonymity of all participants were maintained throughout the study.

### 2.2. Participants

A total of 278 professional caregivers working at care institutions nationwide participated in the survey. To evaluate depression as the primary variable among a group sufficiently exposed to the occupational environment, only professional caregivers with at least one year of work experience were included in this study. Accordingly, 55 individuals with less than one year of experience were excluded, and 223 professional caregivers met the inclusion criteria. Subsequently, one participant with missing data for the dependent variable (depression) and five male participants—given the very small number of males in the sample—were further excluded. Ultimately, 217 professional caregivers were included in the final analysis, of whom 121 (55.7%) were employed full-time and 96 (44.3%) were employed part-time.

### 2.3. Research Variables

The variables were categorized into general characteristics, employment type, and depression. General characteristics were further classified into demographic, work-related, and health-related characteristics.

Regarding demographic characteristics, three variables were included: age, work experience, and income level. Income level was categorized based on the 2022 minimum wage in South Korea, which was 1.91 million KRW per month (calculated using a standard of 209 working hours per month: 8 h per day, 40 h per week). Participants were classified into two groups: those earning less than 1.91 million KRW and those earning 1.91 million KRW or more [17].

Regarding work-related characteristics, eight variables were considered: workplace size, physical burden, health and safety education, preventive programs, rest time, rest facilities, paid leave, and replacement worker support. Workplace size was classified into two categories: fewer than 50 employees and 50 or more employees. Preventive programs were categorized as either sufficient or insufficient, based on whether adequate information regarding health and safety risk factors was provided. Rest time was classified according to whether it was guaranteed in accordance with legal standards (guaranteed vs. not guaranteed). Paid leave was categorized based on whether it could be used freely (use vs. non-use).

Seven health-related variables were included in the analysis: physical activity, meal frequency, presenteeism, subjective health status, fatigue, work-related accidents, and experience of violence. Physical activity was categorized based on the number of days per week participants engaged in exercise intense enough to cause shortness of breath, with two groups defined: fewer than 3 days and 3 days or more. Meal frequency was classified as regular or irregular, depending on whether participants typically consumed three meals per day. Presenteeism was defined as attending work while feeling unwell within the past year and was categorized as either yes or no. Subjective health status was assessed based on participants’ self-perceived overall health and categorized as either good or poor. Fatigue was grouped into three levels according to participants’ usual experience of fatigue: none, average, and fatigued. Work-related accidents were classified based on whether participants had experienced any accident or illness while working in the past year (yes or no). Experience of violence was operationally defined as having encountered at least one of the following forms of abuse in the workplace within the past 12 months: physical violence, verbal abuse, threats, bullying, sexual harassment, unwanted sexual attention, or insulting behavior. This variable was categorized dichotomously (yes or no).

Depression was assessed using the Korean version of the Patient Health Questionnaire-9 (PHQ-9). The PHQ-9 was developed to diagnose mental health disorders in a primary healthcare setting [18], and we used a Korean-translated version [19]. As the Korean PHQ-9 includes fewer items than other depression scales, it requires less time to complete and allows for easy scoring, making it a useful instrument for screening and assessing depression symptoms. The Patient Health Questionnaire-9 (PHQ-9) comprises nine items, each rated on a 4-point Likert scale ranging from 0 (“not at all”) to 3 (“nearly every day”), yielding a total score between 0 and 27. Based on established scoring guidelines, depression severity is categorized as follows: 0–4 points indicate minimal depression, 5–9 points indicate mild depression, 10–14 points indicate moderate depression, 15–19 points indicate moderately severe depression, and 20–27 points indicate severe depression [20]. The 10-point cut-off score of the PHQ-9 has been validated by multiple studies as an appropriate criterion for depression screening, demonstrating high sensitivity, specificity, and strong clinical utility [21,22]. This cut-off is widely used in both research and clinical practice. In this study, a PHQ-9 score of 10 or higher was classified as clinically significant depression, with scores below 10 categorized as low-level depression and scores of 10 or higher as high-level depression. The reliability of the PHQ-9 used in this study was high, with a Cronbach’s α of 0.916.

Employment type was used as a stratification variable and was categorized into two groups: full-time and part-time. In this study, based on the criteria of Statistics Korea, full-time employment was defined as working 36 h or more per week, while part-time employment was defined as wage work with prescribed working hours of less than 36 h per week [23].

### 2.4. Analysis Methods

Data analysis was performed using SPSS version 23.0 in accordance with the study objectives. Frequencies and percentages were calculated to describe participants’ general characteristics (demographic, work-related, and health-related).

The relationships between general characteristics and employment type, as well as the association between employment type and depression, were analyzed using the chi-square test and Fisher’s exact test, as appropriate. To identify factors influencing depression by employment type, logistic regression analysis was performed using a multivariate exploratory approach. To determine the optimal combination of variables, the backward elimination method was used, starting with all variables included in the model and sequentially removing the least significant variables.

All analyses were two-tailed, and statistical significance was set at *p* < 0.05.

## 3. Results

### 3.1. Employment Type and Depression Among Professional Caregivers

Of the 217 participants, 121 (55.7%) were full-time and 96 (44.3%) were part-time. Among them, 59 participants (27.3%) exhibited low depression, whereas 157 participants (72.7%) exhibited high depression (72.7%), indicating a high prevalence of depression among professional caregivers (Table 1).

### 3.2. General Characteristics of Participants by Employment Type

When comparing age distributions by employment type, the proportion of participants aged 55 to 64 years was significantly higher in the full-time group (64.4%), whereas those aged 65 years or older were significantly more prevalent in the part-time group (65.9%). A significantly higher proportion of participants with an income of at least 1.91 million KRT were in the full-time group (72.3%), while a larger proportion with an income of <1.91 million were in the part-time group (57.4%). Regarding place of work, significantly more individuals in the full-time group worked at facilities (86.9%), while a higher proportion in the part-time group worked from home (74.5%). A significantly higher proportion of participants with a workplace size of <50 individuals were in the full-time group (86.9%), whereas those with at least 50 people were in the part-time group (74.5%). A significantly higher proportion of participants working in establishments with fewer than 50 employees were in the full-time group (67.5%), whereas a significantly higher proportion of those working in establishments with 50 or more employees were in the part-time group (58.8%). A significantly higher proportion of participants with rest time were in the full-time group (65.7%), while a higher proportion of those without rest time were in the part-time group (52.5%). The proportion of participants without rest facilities was significantly higher in the part-time group (80.6%) than in the full-time group (54.2%). In addition, significantly more participants with paid leave were in the full-time group (70.8%), while a higher proportion without paid leave were in the part-time group (60.6%). No significant differences were observed between employment types regarding work experience, physical burden, health and safety education, preventive programs, provision of replacement workers, physical activity, meals, presenteeism, subjective health status, fatigue, work accidents, or violence (Table 2).

### 3.3. Depression of Participants by Employment Type

When depression was analyzed by employment type, significant differences were observed in physical burden, health and safety education, preventive programs, rest time, rest facilities, provision of replacement workers, presenteeism, subjective health status, and fatigue for full-time workers. For part-time workers, significant differences were observed in income level, physical activity, presenteeism, fatigue, and violence.

When differences in depression were analyzed for full-time workers, physical burden was significantly higher in the high depression group, with 76.3% reporting difficulties, compared with 46.3% of the low depression group who reported no difficulties. Health and safety education was significantly more likely to be implemented in the low depression group (92.3%), and less likely to be implemented in the high depression group (37.9%). Preventive programs were significantly more likely to be implemented in the low depression group (38.1%) and less likely to be implemented in the high depression group (83.8%). The low depression group was significantly more likely to have rest time (40.0%), whereas the high depression group was significantly more likely to lack rest time (78.6%). Similarly, the low depression group was significantly more likely to have rest facilities (46.0%), whereas the high depression group was significantly more likely to lack rest facilities (78.9%). The low depression group was significantly more likely to have replacement workers provided (41.8%), whereas the high depression group was significantly more likely to have no replacement workers (77.3%). The low depression group was significantly more likely to have no presenteeism (44.78%), whereas the high depression group was significantly more likely to have presenteeism (78.1%). Subjective health status was significantly more likely to be rated as good in the low depression group (41.0%) and as poor in the high depression group (78.6%). The low depression group was significantly more likely to report no fatigue (60.0%), whereas the high depression group was significantly more likely to experience fatigue (78.7%).

When depression differences were analyzed for part-time workers, income level was significantly higher in the low depression group, with 38.5% earning ≥191 million KRW, than in the high depression group, with 84.1% earning <191 million KRW. Physical activity was significantly more frequent in the low depression group, with 30.8% participating in physical activity for ≥3 days, than in the high depression group, where 88.4% participated in physical activities for <3 days. The low depression group was significantly more likely to report no presenteeism (40.0%), whereas the high depression group was significantly more likely to have presenteeism (85.9%). The low depression group was significantly more likely to report no fatigue (45.5%), whereas the high depression group was significantly more likely to experience fatigue (90.2%). The low depression group was significantly more likely to have not experienced violence (29.0%), whereas the high depression group was significantly more likely to have experienced violence (90.9%; Table 3).

### 3.4. Factors Affecting Depression by Employment Type

When factors affecting depression were analyzed based on employment type, significant factors for full-time workers included health and safety education and paid leave, whereas those for part-time workers were physical activity and experience of violence. Full-time workers who did not receive support for health and safety education were 10.731 times more likely to have high depression (95% CI = 1.097–104.952). Full-time workers who could not use paid leave were 6.074 times more likely to have high depression (95% CI = 1.282–28.784).

Part-time workers who performed physical activity for <3 days were 9.748 times more likely to have high depression (95% CI = 1.474–64.482). Part-time workers who had experienced violence were 54.065 times more likely to have high depression (95% CI = 1.715–1703.943; Table 4).

## 4. Discussion

This study examined the factors influencing depression among female professional caregivers according to employment type. The findings confirm that female professional caregivers experience high levels of depression, and that the factors affecting depression differ by employment type. While 72.7% of participants in this study exhibited high levels of depression, previous studies have reported that 15.5% of care workers serving older adults and individuals with disabilities showed moderate or higher levels of depression, 32.3% of home care workers experienced depression, and 26.2% of professional caregivers were found to have clinically significant depression [9,10,11]. The higher rate of depression observed in our study compared to previous studies may be because our survey was conducted in 2022, a period when mental health risk was high due to the COVID-19 pandemic. The global prevalence of depression was 3.44% in 2017 but increased 7-fold to 25% during the COVID-19 pandemic [24]. Based on data from the South Korea Health Insurance Review and Assessment Service, the number of patients with depression increased by 35.1% (an annual average increase of 7.8%) from 691,164 individuals in 2017 to 933,481 individuals in 2021 [25], demonstrating the severity of mental health issues during this period.

Employment type can be categorized as full-time or part-time based on work hours. When comparing the characteristics of workers by employment time, full-time workers generally work for at least 40 h per week and tend to have good employment stability and access to employee benefits. In contrast, part-time workers work based on prescribed hours, often benefit from flexible working hours, but experience lower employment stability and limited employee benefits [9]. When depression was analyzed based on employment type, 68.6% of full-time workers were depressed, while 78.1% of part-time workers reported depression, indicating higher depression levels among part-time workers. Previous studies analyzing depression based on employment type have reported the same findings [14]. High depression levels in part-time workers are caused by low job autonomy, limited social support, and high job instability and needs [14]. These findings may be attributed to the fact that part-time workers, compared to their full-time counterparts, are often required to perform a greater workload within a limited timeframe while lacking adequate institutional support. Such support deficiencies include low wages, insufficient rest facilities, inadequate break time and paid leave, and the absence of substitute personnel, all of which contribute to elevated levels of physical and psychological strain.

When examining the factors affecting depression in female professional caregivers, full-time workers had higher depression levels when they lacked access to health and safety education and could not use paid leave. Job education is important for professional caregivers to perform their duties comfortably and without obstacles. Health and safety education, especially, is of growing importance to protect the health and safety of professional caregivers. However, an inspection of the content of caregiver education reveals that the main focus was on improving clinical ability, with insufficient emphasis on educating professional caregivers about their health and safety [26]. The lack of health and safety education increases anxiety regarding potential workplace accidents and injuries, thereby intensifying the psychological burden associated with insufficient preparedness for such situations. This heightened psychological burden may, in turn, contribute to elevated levels of depression among professional caregivers. A meta-analysis synthesizing studies on psychoeducational and psychotherapeutic interventions for caregivers of older adults reported that such programs are effective in reducing depressive symptoms, with individual interventions demonstrating greater efficacy than group-based approaches [27]. Therefore, to effectively reduce depression among professional caregivers, it is essential to establish a legal framework for the regular and systematic provision of health and safety education. Moreover, the development and implementation of evidence-based programs—such as psychoeducation and cognitive behavioral therapy—that have been empirically proven to alleviate depression are crucial for promoting the physical and mental well-being of professional caregivers [26].

Professional caregivers who could not use their paid leave exhibited a higher level of depression. Among the participants in our study, 66.1% were using paid leave; however, the provision of replacement workers was relatively low, at only 45.5%. The experience of presenteeism, where participants continued working even when sick, was high at 60.8%, demonstrating that professional caregivers were unable to receive support when they needed to use paid leave. Institutional strategies should be implemented to ensure the provision of replacement workers, thereby enabling professional caregivers to take paid leave as needed.

Part-time workers had higher rates of depression when they participated in physical activity <3 days a week and when they had experienced violence.

In this study, 44.8% of part-time workers engaged in physical activity fewer than three days per week. Numerous studies have demonstrated that physical activity alleviates depressive symptoms in individuals with depression and is effective in preventing the onset of depression [28,29]. In particular, aerobic activity has a protective effect against depression, and engaging in regular physical activity has been shown to improve depressive symptoms [30]. Therefore, to support part-time workers in maintaining consistent physical activity in their daily lives, it is essential to provide relevant education and promote participation in community-based programs, such as those offered by public health centers and workers’ health centers.

In this study, 34.4% of part-time workers experienced violence, and among them, 90.9% exhibited symptoms of depression. According to previous research, professional caregivers providing home care for older adults were found to have a 2.3-fold increased risk of depression when exposed to violence, and another study of female workers reported that those who experienced violence had a 4.78-fold higher risk of depression [10,31]. Experiencing violence has been shown to negatively affect not only mental health—such as depression and anxiety—but also physical health, including musculoskeletal pain, headaches, eye strain, abdominal pain, general fatigue, and sleep disturbances [31]. Therefore, it is crucial to establish institutional measures to prevent violence among part-time workers. These measures should include regular education on violence prevention, as well as the guarantee of both rest breaks and rest facilities, so that workers can be separated from perpetrators in the event of violence. In addition, comprehensive support strategies—such as psychological counseling and treatment, as well as community-based support programs for workers who have experienced violence—are also necessary.

## 5. Conclusions

This study has several limitations. First, due to its cross-sectional design, it is limited in its ability to determine causal relationships or temporal sequencing among variables. Nonetheless, cross-sectional studies are valuable for identifying associations between variables and outcomes and for analyzing characteristics in large populations. Future research could address this limitation by employing repeated cross-sectional surveys to examine trends over time or by conducting longitudinal studies to clarify causality more robustly. Second, the study sample consisted solely of female professional caregivers, which limits the generalizability of the findings to male caregivers. Notably, the number of male caregivers in South Korea increased by 21.3%, from 25,350 in 2022 to 30,743 in 2023 [3]. To reflect this demographic change and provide a more comprehensive understanding, future research should include male caregivers. Third, the measurement of violence was operationalized as a binary variable (presence or absence), without accounting for dimensions such as frequency, severity, type (e.g., physical, verbal, sexual), recurrence, or duration. This simplified approach may have resulted in either underestimation or overestimation of its impact on mental health. Future research should adopt more nuanced, multidimensional instruments to better capture the complexity of violent experiences and their psychological consequences. Despite these limitations, this study offers meaningful insights into factors influencing depression among caregivers by employment type. Further research using more rigorous designs and refined measurement tools will enhance the validity and applicability of these findings.

Despite these limitations, this study provides an empirical analysis of factors influencing depression among female professional caregivers by employment type, underscoring the need for targeted policy and practical interventions to enhance mental health among care workers. Future research incorporating male caregivers, utilizing more sophisticated measurement tools, and employing longitudinal designs is warranted to generate stronger evidence for the effective prevention and management of depression in this population.

## Figures and Tables

**Table 1:** Employment type and depression among professional caregivers.

Variables	Categories	N	%
Employment type	Full-time	121	55.7
	Part-time	96	44.3
Depression	Low	59	27.3
	High	157	72.7

**Table 2:** General characteristics of participants by employment type.

Variables	Categories	Full-Time		Part-Time			
		N	%	N	%	X^2^	*p*
Age	<55	25	61.0	16	39.0	14.673	0.001
(years)	55 to <65	76	64.4	42	35.6		
	≥65	20	34.5	38	65.9		
Work experience	<3	28	43.8	36	56.3	7.495	0.058
(years)	3 to <5	25	55.6	20	44.4		
	5 to <10	44	67.7	21	32.3		
	≥10	24	55.8	19	44.2		
Income level *	<1.91	52	42.6	70	57.4	18.989	<0.001
(million KRW)	≥1.91	68	72.3	26	27.7		
Workplace *	Facilities	93	86.9	14	13.1	83.058	<0.001
	Home	28	25.5	82	74.5		
Workplace size *	<50	81	67.5	39	32.5	14.998	<0.001
(employees)	≥50	40	41.2	57	58.8		
Physical burden *	Difficult	80	57.1	60	42.9	0.306	0.668
	Not difficult	41	53.2	36	46.8		
Health and safety education *	Yes	95	55.6	76	44.4	0.014	0.999
	No	26	56.5	20	43.5		
Preventive programs *	Good	84	52.8	75	47.2	2.071	0.167
	Poor	37	63.8	21	36.2		
Rest time *	Yes	65	65.7	34	34.3	7.228	0.009
	No	56	47.5	62	52.5		
Rest facilities *	Yes	50	80.6	12	19.4	21.790	<0.001
	No	71	45.8	84	54.2		
Use of paid leave *	Yes	80	70.8	33	29.2	21.609	<0.001
	No	41	39.4	63	60.6		
Provision of replacement workers *	Yes	55	60.4	36	39.6	1.391	0.269
	No	66	52.4	60	47.6		
Physical activity *	<3	67	60.9	43	39.1	2.397	0.134
(days)	≥3	54	50.5	53	49.5		
Meals *	Regular	71	55.0	58	45.0	0.067	0.889
	Irregular	50	56.8	38	43.2		
Presenteeism *	Yes	73	52.9	65	47.1	1.328	0.256
	No	47	61.0	30	39.0		
Subjective health status *	Good	61	64.0	52	46.0	0.302	0.588
	Not good	60	57.7	44	42.3		
Fatigue	None	10	47.6	11	52.4	1.385	0.500
	Average	50	60.2	33	39.8		
	Fatigued	61	54.0	52	46.0		
Work accidents *	Yes	22	57.9	16	42.1	0.085	0.858
	No	99	55.3	80	44.7		
Violence *	Yes	55	62.5	33	37.5	2.726	0.126
	No	66	51.2	63	48.8		

* fisher’s exact test.

**Table 3:** Depression of participants by employment type.

Variables	Categories	Full-Time						Part-Time					
		Low Depression		High Depression		X^2^	*p*	Low Depression		High Depression		X^2^	*p*
		N	%	N	%			N	%	N	%		
Age	<55	4	16.0	21	84.0	3.815	0.148	3	18.8	13	81.3	0.936	0.626
(years)	55 to <65	28	36.8	48	63.2			11	26.8	30	73.2		
	≥65	6	30.0	14	70.0			7	18.4	31	81.6		
Work experience	<3	9	32.1	19	67.9	0.379	0.945	6	17.1	29	82.9	1.578	0.664
(years)	3 to <5	9	36.0	16	64.0			4	20.0	16	80.0		
	5 to <10	13	29.5	31	70.5			5	23.8	16	76.2		
	≥10	7	29.2	17	70.8			6	31.6	13	68.4		
Income level *	<1.91	16	30.8	36	69.2	0.034	0.999	11	15.9	58	84.1	5.562	0.027
(million KRW)	≥1.91	22	32.4	46	67.6			10	38.5	16	61.5		
Workplace *	Facilities	29	31.2	64	68.8	0.009	0.999	6	42.9	8	57.1	4.107	0.075
	Home	9	32.1	19	67.9			15	18.5	66	81.5		
Workplace size *	<50	22	27.2	59	72.8	2.049	0.211	12	30.8	27	69.2	2.884	0.131
(employees)	≥50	16	40.0	24	60.0			9	16.1	47	83.9		
Physical burden *	Difficult	19	23.8	61	76.3	6.422	0.014	12	20.3	47	79.7	0.282	0.618
	Not difficult	19	46.3	22	53.7			9	25.0	27	75.0		
Health and safety education *	Yes	36	37.9	59	62.1	8.644	0.004	17	22.7	58	77.3	0.065	0.999
	No	2	7.7	24	92.3			4	20.0	16	80.0		
Preventive programs *	Good	32	38.1	52	61.9	5.708	0.020	17	23.0	57	77.0	0.146	0.999
	Poor	6	16.2	31	83.8			4	19.0	17	81.0		
Rest time *	Yes	26	40.0	39	60.0	4.816	0.032	10	29.4	24	70.6	1.642	0.210
	No	12	21.4	44	78.6			11	18.0	50	82.0		
Rest facilities *	Yes	23	46.0	27	54.0	8.426	0.005	5	41.7	7	58.3	3.052	0.129
	No	15	21.1	56	78.9			16	19.3	67	80.7		
Use of paid leave *	Yes	30	37.5	50	62.5	4.071	0.062	11	33.3	22	66.7	3.702	0.070
	No	8	19.5	33	80.5			10	16.1	52	83.9		
Provision of replacement workers *	Yes	23	41.8	32	58.2	5.076	0.031	11	30.6	25	69.4	2.404	0.134
	No	15	22.7	51	77.3			10	16.9	49	83.1		
Physical activity *	<3	20	29.9	47	70.1	0.168	0.698	5	11.6	38	88.4	5.008	0.028
(days)	≥3	18	33.3	36	66.7			16	30.8	36	69.2		
Meals *	Regular	26	36.6	45	63.4	2.169	0.167	14	24.1	44	75.9	0.357	0.619
	Irregular	12	24.0	38	76.0			7	18.9	30	81.1		
Presenteeism *	Yes	16	21.9	57	78.1	6.947	0.014	9	14.1	55	85.9	7.920	0.008
	No	21	44.7	26	55.3			12	40.0	18	60.0		
Subjective health status *	Good	25	41.0	36	59.0	5.239	0.031	13	25.5	38	74.5	0.733	0.462
	Not good	13	21.7	47	78.3			8	18.2	36	81.8		
Fatigue	None	6	60.0	4	40.0	7.690	0.021	5	45.5	6	54.5	10.381	0.006
	Average	19	38.0	31	62.0			11	33.3	22	66.7		
	Fatigued	13	21.3	48	78.7			5	9.8	46	90.2		
Work accidents *	Yes	4	18.2	18	81.8	2.182	0.204	3	18.8	13	81.3	0.126	0.999
	No	34	34.3	65	65.7			18	22.8	61	77.2		
Violence *	Yes	14	25.5	41	74.5	1.657	0.240	3	9.1	30	90.9	4.974	0.036
	No	24	36.4	42	63.6			18	29.0	44	71.0		

* fisher’s exact test.

**Table 4:** Factors affecting depression by employment type.

Variables	Categories	Employment Type					
		Full-Time			Part-Time		
		Adjusted OR	95% CI	*p*	Adjusted OR	95% CI	*p*
Age	<55	1.000			1.000		
(years)	55 to <65	0.243	0.046–1.283	0.096	1.056	0.107–10.391	0.963
	≥65	0.340	0.041–2.834	0.319	3.806	0.284–51.092	0.313
Work experience	<3	0.618	0.115–3.337	0.576	11.435	0.666–196.254	0.093
(years)	3 to <5	0.614	0.097–3.891	0.605	3.323	0.269–41.0170	0.349
	5 to <10	1.606	0.375–6.885	0.524	11.524	0.723–183.663	0.084
	≥10	1.000			1.000		
Income level	<1.91	0.690	0.197–2.420	0.563	2.126	0.275–16.402	0.470
(million KRW)	≥1.91	1.000	0.413–5.078	0.563	1.000		
Workplace	Facilities	3.936	0.569–27.233	0.165	1.553	0.040–61.045	0.814
	Home	1.000			1.000		
Workplace size	<50	2.210	0.626–7.792	0.218	0.308	0.052–1.831	0.195
(employees)	≥50	1.000			1.000		
Physical burden	Difficult	1.363	0.416–4.462	0.609	0.268	0.041–1.739	0.168
	Not difficult	1.000			1.000	0.590–20.565	0.168
Health and safety education	Yes	1.000			1.000	0.257–32.179	0.392
	No	10.731	1.097–104.952	0.041	0.356	0.032–4.003	0.403
Preventive programs	Good	1.000			1.000	0.120–25.050	0.686
	Poor	0.659	0.134–3.234	0.607	0.610	0.041–9.051	0.719
Rest time	Yes	1.000			1.000		
	No	1.399	0.407–4.817	0.594	1.489	0.134–16.542	0.746
Rest facilities	Yes	1.000			1.000		
	No	3.169	0.904–11.102	0.071	10.280	0.400–264.469	0.460
Use of paid leave	Yes	1.000			1.000		
	No	6.074	1.282–28.784	0.023	2.190	0.270–17.783	0.463
Provision of replacement workers	Yes	1.000			1.000		
	No	1.731	0.531–5.644	0.363	3.171	0.439–22.881	0.252
Physical activity	<3	1.227	0.394–3.817	0.724	10.140	1.494–68.800	0.018
(days)	≥3	1.000			1.000		
Meals	Regular	1.000			1.000		
	Irregular	2.021	0.700–5.832	0.193	1.268	0.167–9.637	0.819
Presenteeism	Yes	0.858	0.222–3.313	0.824	8.875	0.742–106.132	0.085
	No	1.000			1.000		
Subjective health status	Good	1.000			1.000		
	Not good	2.012	0.585–6.927	0.268	2.592	0.213–31.478	0.455
Fatigue	None	1.000			1.000		
	Average	3.769	0.523–27.185	0.188	0.728	0.041–13.043	0.829
	Fatigued	4.287	0.607–30.252	0.144	2.208	0.137–35.495	0.576
Work accidents	Yes	0.685	0.115–4.087	0.678	0.484	0.021–11.227	0.61
	No	1.000			1.000	0.088–47.646	0.655
Violence	Yes	1.010	0.277–3.679	0.988	46.384	1.224–1757–505	0.039
	No	1.000			1.000		

## Data Availability

The datasets presented in this article are not readily available because the data was obtained through participation in research conducted by a national institution. Requests to access the datasets should be directed to the corresponding author.

## Abbreviations

The following abbreviations are used in this manuscript:

PHQ-9	Patient Health Questionnaire-9
